# Nurse staffing levels within acute care: results of a national day of care survey

**DOI:** 10.1186/s12913-022-07562-w

**Published:** 2022-04-13

**Authors:** Hannah Hegarty, Thomas Knight, Catherine Atkin, Tash Kelly, Chris Subbe, Daniel Lasserson, Mark Holland

**Affiliations:** 1 Birmingham Acute Care Research Group, Institute of Inflammation and Ageing, University Hospital Birmingham NHS Foundation Trust, University of Birmingham, Edgbaston, Birmingham, B15 2GW UK; 2 Sandwell and West Birmingham NHS Trust, Birmingham, UK; 3 University Hospitals Dorset NHS Foundation Trust, Bournemouth, UK; 4 School of Medical Sciences, Bangor University & Consultant Acute, Respiratory & Critical Care Medicine, Ysbyty Gwynedd, Bangor, LL57 2PW UK; 5 Division of Health Sciences, Warwick Medical School, University of Warwick, Coventry, CV4 7AL UK; 6 Clinical and Biomedical Sciences, Faculty of Health and Wellbeing, University of Bolton, Bolton, BL3 5AB UK

## Abstract

**Introduction:**

The relationship between nurse staffing levels and patient safety is well recognised. Inadequate provision of nursing staff is associated with increased medical error, as well as higher morbidity and mortality. Defining what constitutes safe nurse staffing levels is complex. A range of guidance and planning tools are available to inform staffing decisions. The Society for Acute Medicine (SAM) recommend a ‘nurse-to-bed‘ratio of greater than 1:6. Whether this standard accurately reflects the pattern and intensity of work on the Acute Medical Unit (AMU) is unclear.

**Methods:**

Nurse staffing levels in AMUs were explored using the Society for Acute Medicine Benchmarking Audit 2019 (SAMBA19). Data from 122 acute hospitals were analysed. Nurse-to-bed ratios were calculated and compared. Estimates of the total nursing time available within the acute care system were compared to estimates of the time required to perform nursing activities.

**Results:**

The total number of AMU beds across all 122 units was 4997. The mean daytime nurse-to-bed ratio was 1:4.3 and the mean night time nurse-to-bed ratio was 1:5.2. The SAM standard of a nurse to bed ratio of greater than 1:6 was achieved in 99 units (81.9%) during daytime hours and achieved by 74 units (60.6%) at night.

The estimated time required to deliver direct clinical care was 35,698 h. A deficit of 4128 h (11.5% of time required) was estimated, representing the time difference between the total number of nursing hours available with current staffing and the estimated time needed for direct clinical care across all participating units.

**Conclusion:**

This UK-wide study suggests a significant proportion of AMUs do not meet the recommenced SAM staffing levels, particularly at night. A difference was observed between the total number of nursing hours within the acute care system and the estimated time required to perform direct nursing activities. This suggests a workforce shortage of nurses within acute care at the system level.

## Introduction

Acute Medical Units (AMUs) are a core component of the acute care pathway in the UK [1]. The AMU model has been replicated in other international health care systems [2]. AMUs provide a single location within each hospital dedicated to receiving acutely unwell medical patients deemed to require inpatient care following initial assessment in the Emergency Department or in the community. Care is typically provided for a short period, before either discharge home or transfer to another inpatient ward for admission. The presence of adequate numbers of appropriately trained nursing staff on the AMU is a tenet of safe care. Hospitals with higher nurse-to-patient ratios tend to report better patient outcomes [3–6]. In the UK, national policy makers and regulatory bodies have recognised the link between adequate staffing levels, appropriate levels of training and patient safety [7, 8]. Despite this, concerns regarding nurse staffing levels in acute care persist. Staff shortages are a pervasive problem throughout the health-care system but are particularly prevalent in acute care, where many AMU nursing positions are vacant [9]. Unmanageable workloads and a perceived lack of career progression are cited as causes of low morale and poor retention of senior staff, which perpetuate staffing shortages [10].

Nursing on the AMU provides a unique set of challenges. High rates of patient turnover, in combination with high levels of patient acuity, create a demanding environment enriched for risk. Year-on-year increases in the number of emergency medical admissions and seasonal surges in demand compound this problem [11, 12]. Low staffing levels have been shown to affect the ability of individual nurses to respond to abnormal physiology [13]. Failure to identify the deteriorating patient and escalate care appropriately is a well recognised cause of harm [14]. The Royal College of Nursing (RCN) have raised the concerns of members that inadequate staffing levels across the UK are compromising patient safety and have called for staffing decisions to be based on effective models of care, with coherent workforce planning as opposed to cost and availability [15].

Acknowledgement of the importance of staffing levels has not translated into a clear understanding of what constitutes safe and adequate staffing. The Society for Acute Medicine (SAM) has published relevant guidelines, recommending nurse-to-bed ratios of 1:4 for patients on trolleys and 1:6 for patients in beds [16]. While these recommendations provide a useful standard to facilitate measurement and benchmarking, the use of fixed staffing ratios in isolation is problematic. Nurse to bed ratios do not take into account the pace of work driven by patient flow, the skill-mix of the team or relevant patient factors, all of which are likely to influence the number of nurses required. The National Institute for Health and Care Excellence (NICE) have provided specific guidance to aid decision making in relation to nurse staffing levels, where the time required to complete common patient orientated tasks is estimated, to allow a more accurate prediction of the number of staff required [17]. However, these guidelines are based on expert opinion without the benefit of robust empirical evidence.

The Society for Acute Medicine Benchmarking Audit (SAMBA) has run annually since 2012, and by convention takes place on the penultimate Thursday in June [18]. SAMBA records patient level data from individual clinical records to assess performance across a range of pre-specified criteria related to acute medical care. The audit combines organisational and patient level data to provide a detailed picture of how acute medical care is delivered in the UK. We used data from the 2019 SAMBA (SAMBA19) to better understand the provision of nurse staffing resources within the AMU and the skill-mix of the nursing workforce. Our aim was to describe the national nursing staffing levels currently present on AMUs in the UK, including evaluation of skill-mix. We also looked to and compare staffing levels to estimates of the total time required to provide nursing care to patients admitted to the AMU during the study period. This is the first time data on nurse staffing levels has been reported using SAMBA.

## Methodology

Recruitment to SAMBA19 was open to all hospitals in the UK receiving acutely unwell (non-elective, adult) medical patients. Non-acute and community hospitals were excluded. SAMBA19 was undertaken on Thursday 27th June 2019, between 00:00 and 23:59. SAMBA19 was the first SAMBA survey to collect data regarding nurse staffing levels and skill-mix.

SAMBA is a cross-sectional audit with an observational design. Patients aged 16 years or above who were seen for either admission or assessment as part of the internal medicine admission process (‘take’) or ambulatory emergency care (AEC) were eligible for inclusion. Patient level data collection was performed voluntarily by local clinicians, overseen by a named responsible consultant physician. The SAMBA protocol is publicly available [19].

Prior to the collection of patient level data, a lead acute medicine clinician at each participating AMU completed an online questionnaire to provide organisational and unit level data, relating to staffing levels on the AMU. Data on staffing levels could only be submitted once from each AMU. The questions regarding nursing numbers, level of nursing experience and the specific roles performed by staff were developed by a group of nurses affiliated with SAM. Total number of nurses present on the AMU during the 24-h data collection window was measured, with nurse-to-bed ratios during daytime (08:00–19:59) and nighttime (20:00–07:59) hours calculated using the reported number of available beds within each AMU (occupied and unoccupied). The number of non-registered nurses, band 5 nurses, band 6 nurses and band 7 or above nurses were recorded separately [20].

The proportion of units which achieved the SAM standard was calculated. In order to determine whether the use of non-registered nurses was higher in units with fewer registered nurses, the ratios of registered to non-registered nurses were compared between units with nursing levels above and below the recommended SAM standard.

The unit and patient level data collected were used to generate an estimate of the total number of nursing hours available within all contributing AMUs. This estimate was compared with an estimate of the anticipated time required to complete nursing tasks informed by the relevant NICE guidance on safe staffing levels [17]. The total number of available AMU beds taken from the unit level data were combined with the total number of admissions to each AMU recorded at the patient level.

To estimate the amount of nursing time required to complete tasks at the system level. This was achieved by multiplying the anticipated number of occupied beds, or the number of AMU admissions, by the proportion of patients deemed likely to require each nursing intervention and the time the time taken to perform each nursing intervention as stated by the NICE guidelines.

These estimates were made under multiple assumptions. A bed-occupancy rate of 90% was used to inform the estimate, consistent with national averages during the study period [21]. It was anticipated that patients with features of frailty would have increased nursing needs. Patients with features of frailty were anticipated to require complex admission assessment and discharge planning. The prevalence of frailty was approximated at 50% based on previous evidence [18, 22]. The frequency of physiological observations was based on the distribution of NEWS2 values obtained on admission, combined with national guidance on the frequency of observation [31].

All admitted patients were assumed to require administration of medications (including assessment of whether ‘as required’ medications were needed), 80% were assumed to require administration of intravenous medication [23]. Intravenous fluid therapy was felt likely to be required in 50% of patients [24] (rate of 8 hourly in 30%, > 8 hourly in 10% and complex fluid management involving hourly fluid balance in 10%) [25]. All patients were deemed to require some form of direct communication each day (routine in 50% and significant in 50%). Consensus opinion as to whether these assumptions reflected clinical experience was obtained by discussion (HH, TK, CA), and disagreements reviewed by all authors. The assumptions made for individual tasks are provided in Table 1.

**Table 1:** Estimates of time required to provide direct clinical nursing care within units participating in SAMBA19

Activity	Approximate time per task	Frequency of task of task in 24 h period	Estimate of applicable patients (% = proportion estimated to require task)	Total attributable time (hours)	Task appropriate for non-registered staff appropriate
**Admissions (*n*** **=** **6423)**					
Admission assessment	20 mins	Once	3212	1070	No
Complex admission assessment*)*	30 mins	Once	3211	1605	No
**Discharge planning / inter hospital transfer (*n*** **= 3372) (based on AMU turnover 75% and bed occupancy 90%**					
Simple follow up and transfer home	10 mins	Once	2530 (75%)	421	No
Coordination of different services	20mins	Once	749 (20%)	225	No
Organizing complex services, support and equipment	30mins	Once	126 (5%)	63	No
**Patient escort (based on 5% of AMU patients requiring escort during AMU stay)**					
Radiology / endoscopy	30 mins	Once	224	112	No
**Procedures, treatments and end-of-life care (based on number of AMU beds and 90% occupancy)**					
Simple wound dressing, specimen collection	10 mins	Once	4048 (90%)	674	Yes
Nasogastric tube insertion. Catheterisation, Multiple simple dressings	20 mins	Once	249 (5%)	75	No
Complex wound management	30 mins	Once	50 (1%)	22	No
Care after death	30 mins	Once	250 (2.5%)	56	No
**Observations (*n*** **= 4997) (based on AMU bed numbers, NEWS2 [**31**] and bed occupancy 90%)**					
Routine (4 to 6 hourly)	10 mins	Four	3598 (NEWS2 < 5)	2399	Yes
Increased (2 to 4 hourly)	10 mins	Six	674 (NEWS2 5–6)	674	Yes
> 2 hourly	10 mins	Twelve	224 (NEWS2 ≥ 7)	450	Yes
**Medication administration (based on AMU bed numbers and bed occupancy 90%)**					
Regular oral	10 mins	Four	4498	2998	No
IV or frequent PRN	20 mins	Three	3598 (80%)	3598	No
Medication requiring complex preparation (2 nursing staff) eg controlled drugs	30 mins	Two	404 (10%)	448	No
**Direct contact and communication (based on AMU bed numbers and bed occupancy 90%)**					
Routine	10 mins	Once	2249 (50%)	375	No
Significant	30 mins	Once	2249 (50%)	1125	No
**Eating and drinking (based on AMU bed numbers and 90% bed occupancy)**					
Assistance	20mins	Three	2263 (50%)	2263	Yes
Parenteral	30mins	Three	49 (1%)	67	No
**Fluid management (based on AMU bed numbers and 90% bed occupancy)**					
8 hourly IV fluids	10 mins	Three	1357 (30%)	679	No
IV fluid > 8 hourly or blood products	20 mins	Four	452 (10%)	602	No
Complex fluid management (such as hourly fluid balance)	10 mins	Twenty-four	899 (10%)	1810	No
**Management of equipment (based on AMU bed numbers and 90% bed occupancy)**					
Simple intermittent (catheter, IV access)	10 mins	Once	4273 (95%)	712	No
Drains / stomas / central lines	20 mins	Three	225 (5%)	225	No
Ventilatory support (including NIV)	30 mins	Twenty-four	44 (1%)	539	No
**Mobilisation (based on AMU bed numbers and 90% bed occupancy)**					
Assistance needed	20 mins	Four	1799 (40%)	2399	Yes
Assistance of two nursing staff required	30 mins	Four	449 (10%)	899	Yes
**Skin and pressure areas care (based on AMU bed numbers and 90% bed occupancy)**					
2–4 hourly	20 mins	Five	449 (10%)	831	Yes
More frequently than 2 hourly	30 mins	Twelve	250 (5%)	1350	Yes
**Toileting (based on AMU bed numbers and 90% bed occupancy)**					
Assistance needed	20 mins	Four	1799 (40%)	2399	Yes
Frequent assistance of > 2 nursing staff required	30 mins	Four	449 (10%)	899	Yes
**Washing, bathing and dressing (based on AMU bed numbers and 90% bed occupancy)**					
Assistance with some hygiene needs by 1 member of nursing staff	20 mins	Four	2249 (40%)	2398	Yes
Assistance with all hygiene needs or needing 2 nursing staff	30 mins	Four	449 (10%)	899	Yes
				**Hours required to perform all activities**	**Hours required to perform activities appropriate non-registered nurses**
				35, 361	18, 534

The administration of oral and parenteral medications and clinical tasks associated with care coordination were deemed appropriate for registered nursing staff in accordance with NMC guidance [26]. Tasks suitable for non-registered nurses were identified and summated separately. The number of nursing hours required to meet the demands of nursing care on the day of SAMBA was compared with the actual number of nursing hours available recorded by individual units. It was assumed that matrons and ward managers did not undertake direct clinical care, due to other responsibilities.

## Results

SAMBA19 collected paired unit and patient data from 130 AMUs. Complete data relating to nurse staffing and workforce planning was provided by 122 AMUs (93.8%), with individual patient level data collected for 5849 patients seen through these units, 8 AMUs did not provide data on nurse staffing levels and were not included in the analysis.

The most common working pattern, employed by 70 units (57.4%) was exclusive 12-h shifts. A combination of 12-h and 8-h shifts were implemented in 51 units (41.8%). Only one unit used solely 8-h shifts. During the daytime, 1159 nurses were on duty; this included 913 staff nurses and 246 senior nurses (ratio 3.7:1). At nighttime, 970 nurses were on duty; this included 801 staff nurses and 169 senior nurses (ratio 4.7:1).

### Nurse staffing levels

**Fig. 1:**
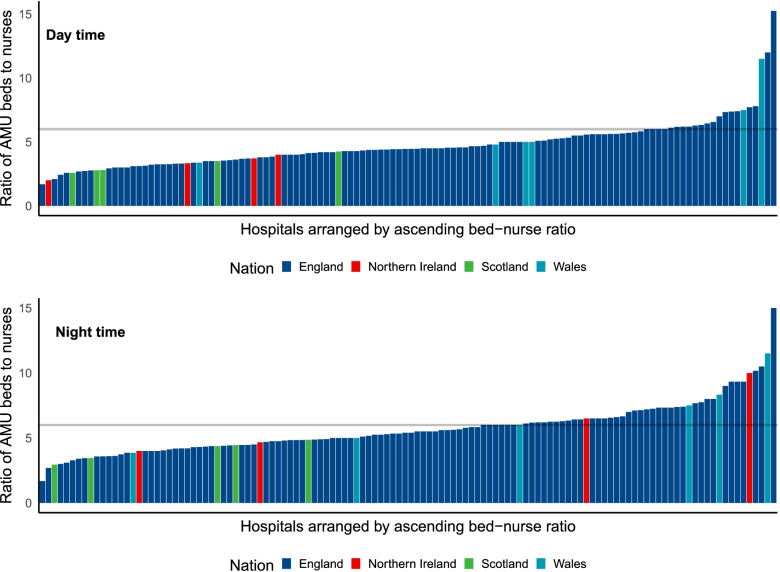
Variation in AMU bed to nurse ratio by country. *hospitals ordered by increasing bed to nurse ratio. Horizonal line represents SAM standard (6:1)

**Fig. 2:**
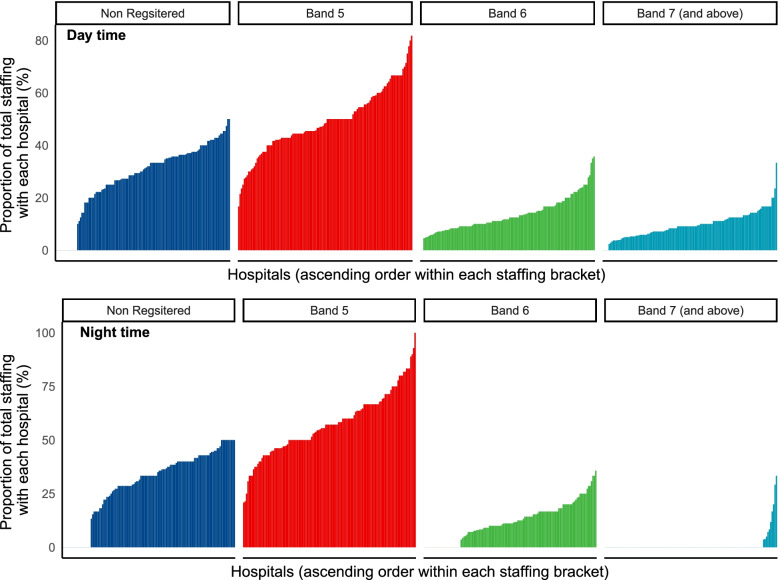
Bar plots demonstrating variation in skill-mix at the hospital level

Nurse-to-bed ratios were calculated. The total number of AMU inpatient beds across all 122 units was 5028. The mean daytime nurse-to-bed ratio was 1:4.7 and the mean nighttime nurse-to-bed ratio was 1:5.7). The SAM standard of a nurse-to-bed ratio of greater than 1:6 was achieved in 104 units (85.2%) during daytime hours and achieved by 80 units (65.6%) at night. The variation in nurse to bed ratios at day and night is provided in Fig. 1 stratified by UK country. The variation in skill-mix during the day and night periods is presented in Fig. 2. The Band 5 nurses represented on average 48.9% (range 16.7–81.2%) of the of the nurse staffing workforce during the day and 57.4% range (20.8–100%) at night within participating hospitals.

**Fig. 3:**
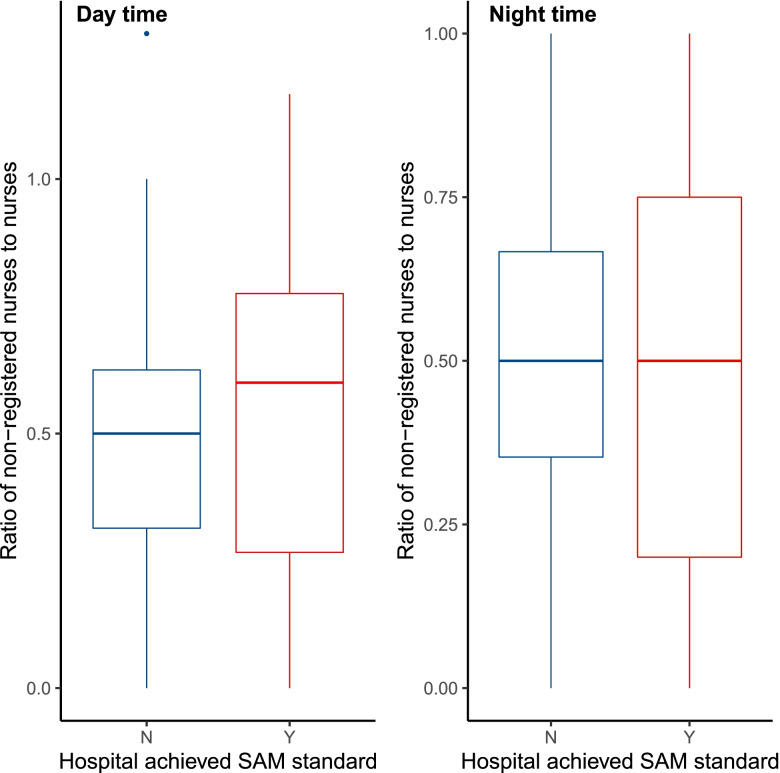
Boxplots comparing the ratio of non-registered to registered nurses at day and night stratified by achievement of the SAM standard in relation to nurse to bed ratio

The number of non-registered nurses on duty during the evaluation period was recorded. During the day, 562 non-registered nurses were on duty, a ratio of registered to non-registered nursing nurses of 2.1:1. At night, 466 non-registered staff were on duty, a ratio of registered to non-registered nurses of 2.1:1. In AMUs with staffing levels above the SAM standard these ratios were 2.1:1 and 2.1:1 during the day and night respectively. The corresponding ratios in AMUs with staffing levels below the SAM standard were 1.8:1 and 1.5:1. The ratio of non-registered to registered nurses at daytime and night time is provide in Fig. 3.

### Numbers of hours in the system

The total amount of registered nursing time available during the 24-h evaluation period was 22,344 h, assuming 75% of the total workforce were working a standard 12-h shift and the remainder 8-h shifts (assuming mandated break periods were observed). The total amount of non-registered nursing time was 10,280 h, assuming the same shift pattern.

The total amount of time required to complete direct nursing tasks was estimated. The calculations and assumptions used to derive this estimate are provided in Table 1. In total, 4997 available AMU beds were recorded in SAMBA equating to 4497 occupied beds (assuming a bed occupancy of 90%). A summary of the estimates and their derivation is provided in Table 1. The estimated time required to deliver direct clinical care was 35,361 h, including 16,827 h of activities only appropriate for registered nurses and 18,534 h of tasks appropriate for non-registered nurses. There was a surplus of 5517 h comparing the number of hours required to complete tasks only appropriate for registered nurses with the number of available registered nurse hours. There was a deficit of 8255 h comparing the number of hours required to complete non-registered nurses and the number of available non-registered nurse hours.

There was a total deficit of 2738 h between the estimated time required to deliver all care and the actual time available. An additional 238 non-registered nursing staff working 12 h shifts would be required to meet the predicted deficit.

## Discussion

SAMBA19 provides insight into nurse staffing levels in acute medical care at the system-level. The results suggest a short-fall in staffing levels within the 24-h study period. SAM recommend a nurse to bed ratio of 1:6, but this was not achieved in approximately 20% of AMUs during daytime hours. Performance against the standard was considerably poorer at night. Nighttime staffing was also characterised by a lower ratio of band 6 or higher nurses to more junior colleagues.

Estimates of the time required to perform direct clinical tasks, based on current NICE guidelines, suggest a significant gap between the time required to deliver care and the total number of nursing hours available. These estimates only calculate the nursing time required for direct clinical care. The provision of direct clinical care does not include time between tasks or documentation, which represent a significant proportion, if not the majority, of time used in delivering nursing care. A previous observational study using a validated tool to measure working patterns and the time distribution of nursing tasks suggested only a fifth of available time was devoted to activities involving direct patient contact [27]. The study findings did not account for the additional nursing time required to provide high dependency level care to those at the severest end of the acuity spectrum with conditions such as septic shock, respiratory failure requiring non-invasive ventilation, or diabetic ketoacidosis. These conditions are relatively common on the AMU and place significant additional demands on nursing staff. Furthermore, these study findings do not account for nursing time devoted to bed coordination; this is a complex and time consuming role often designated to a member of the nursing team. The ability to perform direct clinical activity is likely to be severely limited when undertaking the coordination role. Combining all these factors together, nurse staffing levels in AMUs are under-resourced.

Nurse to bed ratios in isolation are recognised as a poor measure for staffing requirements as they do not adequately account for the multitude of factors that determine whether demand and supply are adequately matched [28, 29]. NICE recommend the use of an in-depth analysis of demand and skill-mix incorporating a time task approach to ensure safe staffing levels [17]. Nursing levels should be matched to the acuity and complexity of the patients and to the pace of the work dictated by patient flow. In this regard, staff planning requires consideration of patient factors, the staffing skill-mix in terms of experience and competencies, and ward factors that relate to the process of care and added responsibilities. Patients managed on AMU, particularly during the first 24-h, are at the peak severity of their illness [30]. This is the point at which the need for medical intervention and intensive monitoring is highest and the probability of deterioration greatest. Many patients are reaching the end-of-life and in these circumstances compassionate nursing care requires time to ensure both patients and relatives receive appropriate support. The changing nature of acute medical admissions, with an increase in multiple co-morbidities and frailty, also creates pressures on the time required to provide quality care. It is not unusual for patients on the AMU to require 1:1 care to ensure their safety. The variable nature of acute care can make workforce planning challenging. The results of this study raise concerns as to whether current staffing levels are adequate for the demands placed on nursing staff to provide quality care. This deficiency in staffing has the potential to materially affect outcomes and the experience of care by acute medical patients.

The deficit in the number of hours to provide care was driven by tasks that could be appropriate for non-registered nurses. Classification of tasks on this basis is a simplification of reality. It would be expected that a proportion of a registered nurses time would involve delivering basic care in addition to more complex tasks which would be inappropriate for non-registered colleagues. Although our estimates suggest a surplus of hours to perform tasks only appropriate for registered nurses, this did not account for the indirect time required to perform these tasks, such as documentation, collecting of equipment and communicating with other members of the medical team. Not accounting for this indirect time is likely to have led to a significant under-estimate of the time required to conduct these tasks. Increasing the number of non-registered nurses may help reduce some of the gap between anticipated workload and available staff but is unlikely to provide a solution to inadequate staffing levels in isolation. High ratios of non-registered to registered nurses have been linked to higher mortality during in-patient care [31]. The development of new roles such as nursing associates, with enhanced spectrum of practice, may help provide additional nursing care while also performing some tasks traditionally only performed by registered nurses [32].

The NHS staff survey highlighted the relationship between overstretched staff with excessive workloads and poor morale [33]. These workforce factors are known to affect patient care [6, 34]. A work environment in which staff feel overstretched and without the necessary resources to provide high quality care leads to stress and staff burnout [35]. In the NHS, nursing positions in acute medical care have the highest vacancy rates [9]. High staff turnover and poor retention inhibits the passage of knowledge from senior staff to those less experienced. The principles laid out in the Interim NHS People Plan to increase the intake of new nurses and ensure appropriate career progression should be implemented urgently [36].

### Strengths and limitations

We provide a system-wide estimate of the mis-match between nurse staffing levels and the time required to deliver basic nursing care. We used broad generalisations which are likely to under-appreciate variation between AMUs. The assumptions used to estimate the total number of nursing hours required to complete direct clinical care are likely to lack precision. The proportion of patients predicted to require each intervention was based on a subjective assessment informed by staff nurses and clinicians with experience working in acute work. The same is true of the estimates of time required to complete tasks, although these values were taken directly from the relevant NICE guidelines. The estimates within the NICE guidelines may not accurately represent reality. While these limitations are likely to affect the accuracy of our findings, the large disparity in the estimate of available versus required nursing time to deliver acute medical care supports the conclusion that current nursing levels are insufficient. We used conservative estimates in relation to bed occupancy which may have underestimated demand. The single day-of-care methodology employed by SAMBA does not account for seasonal and diurnal variation or the weekend affect which are known to affect demand on the AMU [37].

Our findings assume all nursing time is devoted to direct clinical care, this ignores the time required to document case-records, find equipment or undertake unexpected tasks. Therefore, we contend that any inaccuracies in our study findings underestimate the extent of the problem. Our findings provide a system level description of nurse staffing levels at the system level but our study design precludes meaningful analysis of how nurse staffing levels impact on clinical care, patient experience and outcomes.

There are approximately 225 AMUs within the UK; our evaluation therefore represents 58% of AMU’s within the NHS [38]. There is no obvious reason to assume nursing staffing levels within non-participating units would be significantly different. Our findings are relevant, with important implications, even if not generalisable to the entire system.

## Conclusion

This UK-wide study suggests that despite reasonable compliance with current SAM staffing levels, when based on a nurse per bed ratio, in reality acute medical care faces a staffing crisis among nurses. There is not enough nursing time within the system to complete direct clinical tasks, as predicted by our study findings. Ultimately our findings highlight the need for an increase in the number of nurses working in AMUs. A concerted effort to recruit and retain nursing staff is urgently required. A valid fear would be declining quality in patient care, especially if the current situation deteriorates further.

## Data Availability

The datasets used and analysed during the current study are available from the corresponding author on reasonable request.
